# Implementation and evaluation of an e-health innovation for personalized care for patients with amyotrophic lateral sclerosis (ALS): protocol for a participatory action research study

**DOI:** 10.1186/s43058-021-00130-z

**Published:** 2021-02-25

**Authors:** M. L. Dontje, E. Kruitwagen - van Reenen, J. M. A. Visser-Meily, A. Beelen, on behalf of the Study Group ALS Home-monitoring and Coaching

**Affiliations:** 1 Department of Rehabilitation, Physical Therapy Science and Sports, UMC Utrecht Brain Centre, University Medical Centre, Utrecht, the Netherlands; 2 Centre of Excellence for Rehabilitation Medicine, UMC Utrecht Brain Centre, University Medical Centre Utrecht, and De Hoogstraat Rehabilitation, Utrecht, the Netherlands

**Keywords:** Implementation science, Telemedicine, Rehabilitation, Amyotrophic lateral sclerosis, Action research

## Abstract

**Background:**

In the absence of a cure for amyotrophic lateral sclerosis (ALS), a progressive neurodegenerative disease, treatment consists of symptomatic management by a multidisciplinary healthcare team and is mainly aimed at optimizing patients’ quality of life. Because the course of the disease is often erratic and varies between patients, it is imperative for patients with ALS to be closely monitored. E-health innovations that can monitor disease progression remotely have great potential to tailor the care to the needs of individual patients with ALS. Therefore, the e-health innovation “ALS Home-monitoring and Coaching” was developed employing a user-centered design process and implemented at the University Medical Center Utrecht, the Netherlands in 2017. Because ALS Home-monitoring and Coaching was shown to be feasible and well received by patients and healthcare professionals at University Medical Centre Utrecht, we aim to implement this e-health innovation nationwide, starting with 10 ALS care teams in different rehabilitation settings spread across the Netherlands.

**Methods:**

This research focuses on the implementation process and the user experiences with ALS Home-monitoring and Coaching of both patients and healthcare professionals. We will use a participatory action research approach, with the stakeholders involved in all stages of the implementation process. The implementation process model of Grol and Wensing was used to structure and support planning, execution and evaluation of the implementation strategy. The expected barriers and facilitators will be explored and identified in focus group settings using the Theoretical Domains Framework. After that, each team will develop their own action plan with strategies for how to resolve each barrier. The teams will include 5-10 ALS patients with whom they will test their implementation plan and provide care with ALS Home-monitoring and Coaching for approximately 3 months. Afterwards, the implementation and the user experiences will be evaluated with digital surveys based on the evaluation framework of Proctor (e.g., acceptability, adoption, appropriateness).

**Discussion:**

Using implementation theories, this study will provide inside in factors influencing implementation outcomes and strategies that can be used to overcome barriers. This will enhance our understanding of how to successfully implement e-health innovations in multidisciplinary care in rehabilitation settings.

**Trial registration:**

Trial NL8542 registered at Netherlands Trial Register (trialregister.nl) on 15th April 2020.

Contributions to the literature

- To increase the possibility of a successful implementation, it is important to not “reinvent the wheel again”, but to build on existing implementation knowledge as much as possible. Therefore, we used implementation theories to plan, execute and evaluate the implementation strategy.
- Involving stakeholders in all stages of implementation is recommended; therefore, we combined the implementation theories with a participatory action research approach.
- It is anticipated that this study will contribute to our understanding of how e-health innovations can successfully be implemented in multidisciplinary care in rehabilitation settings, which may support other implementation initiatives as well.

## Background

### Context

Amyotrophic lateral sclerosis (ALS), progressive spinal muscular atrophy (PMA), and primary lateral sclerosis (PLS) are closely related progressive neurodegenerative diseases for which no known cure exists yet. In this paper, we use ALS as umbrella term for ALS, PMA and PLS. Treatment consists of symptomatic management by a multidisciplinary healthcare team and is mainly aimed at optimizing patients’ quality of life and prolonging survival [1, 2]. Because the course of the disease is often erratic and varies between patients, it is imperative for patients with ALS to be closely monitored in order for the multidisciplinary health care team to be able to provide the best care possible. Currently, patients are required to visit the clinic usually every 3–4 months for their check-ups by the multidisciplinary health care team situated within a rehabilitation center or hospital. However, patients are not being monitored between visits, which can have detrimental effects on the continuity of multidisciplinary care these patients need. Moreover, for many patients with ALS, it can be difficult to visit their healthcare team, due to having difficulties with traveling (long distances) and the amount of energy it costs to spend long days at the clinic [3, 4]. To better tailor the care to the needs of individual patients and to lessen the burden for patients of having to travel to the clinic and attend long and exhausting clinic days, e-health innovations that can monitor disease progression remotely have great potential [5].

### ALS home-monitoring and coaching

Therefore, an e-health innovation called “ALS Home-monitoring and Coaching” was developed in co-creation with patients with ALS, caregivers, healthcare professionals, managers and information technologists. The development of ALS Home-monitoring and Coaching was a cyclic process which took into account the needs of the end-users with a user-centered design approach [6].

**Fig. 1:**
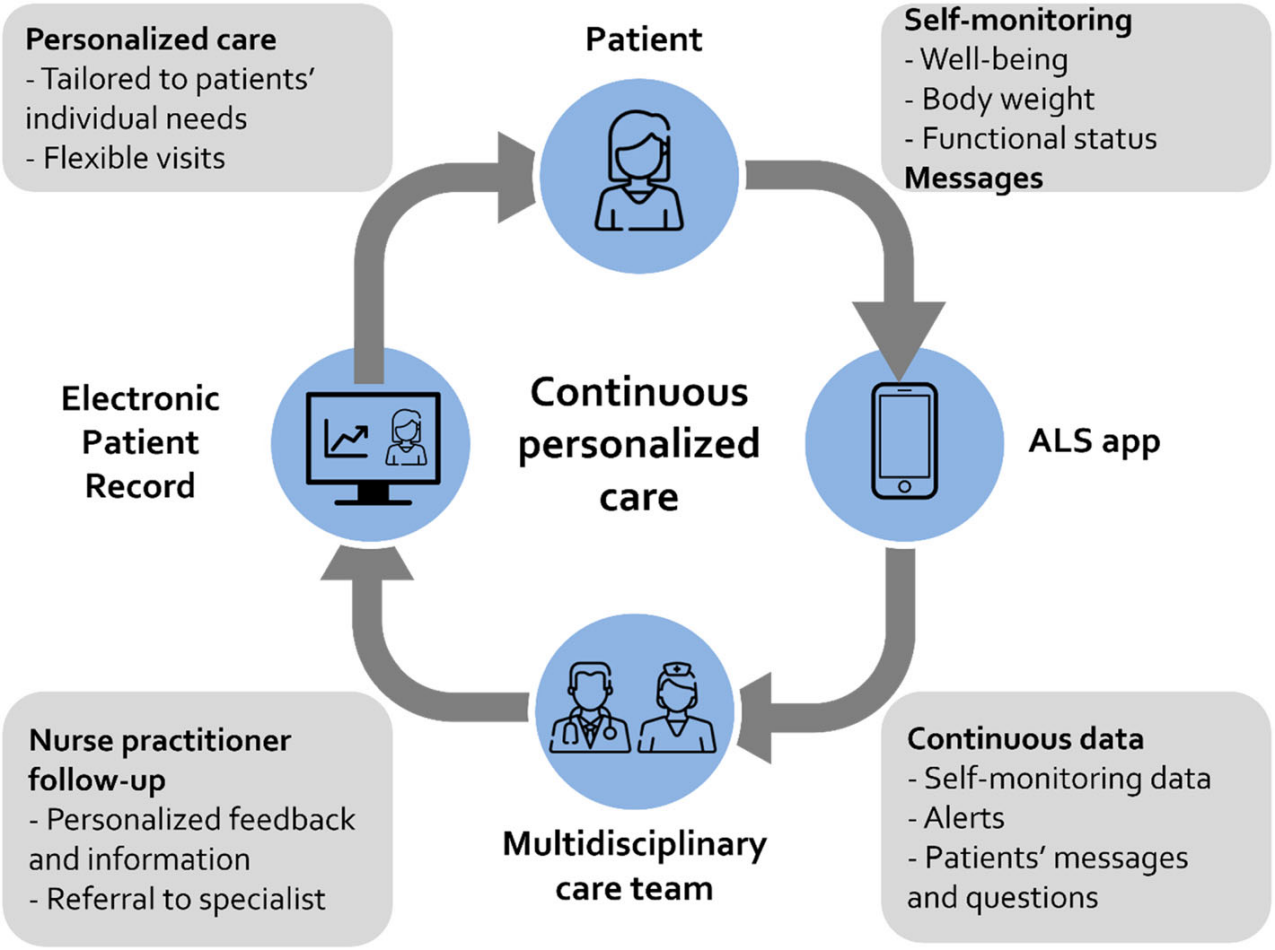
The key features of ALS Home-monitoring and Coaching (adapted from Figure 1, Helleman et al. [5])

In short, with this e-health innovation, patients can keep track of their well-being, body weight and functional status with a mobile application, and their measurements are being monitored by a healthcare coach (i.e., a nurse practitioner or occupational therapist) following a standardized protocol based on ALS guidelines. The key features of ALS Home-monitoring and Coaching are shown in Fig. 1.

### Self-monitoring with a mobile app

The self-monitoring app (Philips Vital Health, the Netherlands) can be downloaded from Google Play and the App Store and can be used on a smartphone and a tablet. A web-based version of the app can be used on a personal computer. After the healthcare coach has created an account for a patient, the patient can use the app to monitor their own health status, by assessing their well-being, body weight, and functional status. To assess well-being, patients are asked to answer the question “How are you today?” with a score from 1 to 10. Additionally, they can elaborate on the score by adding a comment in the app. To assess body weight, patients are asked to weigh themselves and to enter the data in the app. To assess functional status, patients are asked to complete the Amyotrophic Lateral Sclerosis Functional Rating Scale-Revised (ALSFRS-R) in the app [7, 8]. The questionnaire consists of 12 questions measuring the extent to which patients are capable and independent in performing 12 functional activities, e.g., walking, swallowing, and writing. Answer categories range from [4] no loss of function to [1] total loss of function. The default setting for the frequency of measurements is daily for well-being, weekly for body weight, and monthly for functional status. The frequency of the measurements can be adjusted at the discretion of the patients. Completed measurements will automatically be transmitted to a central server, from where it can be viewed by the healthcare professionals of the ALS care team. Patients can always view their own data in the app.

### Alerts

The e-health program has an alert system built in that notifies the healthcare coach whenever there is a significant deterioration in well-being or body weight. The healthcare coach monitors these alerts and discusses them with the multidisciplinary care team when necessary.

### Message function

The message function within the app allows patients and the healthcare coach to contact each other in an easily accessible way. Patients can use the message function to discuss and ask questions about, e.g., symptoms and treatment options, but the message function can also be used to ask for a consultation with one of the healthcare providers of the ALS care team. The healthcare provider aims to respond to questions within three working days. Although the messages will be regularly checked by one of the healthcare providers, the message function is not suitable for emergencies. This is explained to the patients when they start using the app.

### Monthly monitoring by healthcare coach

A healthcare professional from the multidisciplinary ALS care team who is trained in monitoring and coaching for ALS Home-monitoring and Coaching (i.e., nurse practitioner, occupational therapist) checks and follows up on the alerts and messages and evaluates the individual health status data of the patients. This healthcare coach will always work closely together with the physiatrist and uses a standardized monitoring and care protocol based on ALS guidelines. The protocol specifies the correct response to specific changes in functioning, what topics should be discussed with one of the other healthcare professionals of the multidisciplinary care team, and if and when a referral to one of the other healthcare professionals for a face-to-face consultation is necessary. Each month, the healthcare coach sends a message in the app with personalized feedback and they have the opportunity to share other additional relevant information via the app with the patient.

### Feasibility and user experiences

After a small successful pilot study (*N* = 10), ALS Home-monitoring and Coaching was implemented in the ALS clinic of the University Medical Centre Utrecht, the Netherlands, in 2017, where it is now fully integrated in the usual care for patients with ALS [9]. After it had been used for approximately 18 months, Helleman et al. evaluated the user experiences with ALS Home-monitoring and Coaching [5]. They concluded that the adoption rate was high; 80% of the patients choose for ALS Home-monitoring and Coaching. They also found that the adherence was good, especially for the weekly body weight measurements and the monthly functional status measurements. The vast majority of patients (83% and 87%, respectively) completed ≥ 50% of the agreed-upon body weight measurements and ≥ 75% of the agreed-upon functional status measurements. Even though some patients experienced some technical problems with logging on and some did not like being confronted by decreasing ALSFRS-R scores, the user experiences were mainly positive. The vast majority of patients perceived ALS Home-monitoring and Coaching as positive, helpful, easy, not time-consuming, and not burdensome and almost everyone would recommend other patients to use ALS Home-monitoring and Coaching. They experienced more control over care and valued the flexible consultations. Helleman et al. showed that healthcare professionals were mainly positive about ALS Home-monitoring and Coaching as well [5].

### Study focus: implementation

E-health, and especially remote monitoring, is still relatively new in the world of rehabilitation. Because ALS Home-monitoring and Coaching was shown to be feasible and well received by patients and healthcare professionals at the University Medical Centre Utrecht, we aim to implement this e-health innovation nationwide, starting with 10 ALS care teams in different rehabilitation settings (university hospital, regional hospital, rehabilitation center), spread across the Netherlands. This research focuses on the implementation process and the user experiences of both patients and healthcare professionals with ALS Home-monitoring and Coaching. We will use a participatory action research approach and an implementation process model to implement, an implementation determinant framework to understand what influences the implementation outcomes in our project, and an evaluation framework to evaluate the implementation and the user experiences (described in more detail below). The main research questions are as follows: (1) Can ALS Home-monitoring and Coaching successfully be implemented in different rehabilitation settings? (2) What are the most common facilitators and barriers for implementing ALS Home-monitoring and Coaching in a rehabilitation setting? (3) Which implementation strategies are most successful in which context? (4) To what extent is ALS Home-monitoring and Coaching feasible, acceptable, and usable for patients and healthcare professionals?

## Methods/design

### Ethics and study registration

Ethical approval for this study was waived by the Research Ethics Committee of the University Medical Centre Utrecht (20-204/C), and the study is registered in the Netherlands Trial Register (NL8542, 2020-04-15).

### Implementation settings

The total number of patients with ALS in the Netherlands is approximately 1500. The care for these patients is divided over 38 multidisciplinary ALS care teams of rehabilitation centers that are affiliated with the ALS Care Network. The ALS Care Network is a nationwide healthcare network with a mission to provide the best care possible for patients with ALS, PMA, or PLS. In the Netherlands, the settings in which those ALS care teams operate vary from small regional hospitals, to large university medical centers, to rehabilitation centers. The settings vary not only in the number of patients in care, but also in organizational and financial structures and resources. For this project, 12 ALS care teams were selected based on their setting (university hospital, regional hospital, rehabilitation center), geographical location in The Netherlands (urban/rural), and number of patients with ALS/PMA/PLS in care of whom 10 consented to participate.

Each participating center will compose of a project team, consisting of the main stakeholders including the physiatrist(s) of the ALS care team; two or three allied health professionals (i.e., one of them will become the healthcare coach, and one allied health professional will fulfill the role of knowledge broker); the manager of the rehabilitation center or department; one scheduler; someone who will become the administrator of the platform and can provide technical assistance if necessary; one or two ALS/PMA/PLS patients; and one or two informal caregivers. The project team will be informed of the details of ALS Home-monitoring and Coaching and the implementation process.

### Implementation strategy

The implementation strategy we have chosen for this project is guided by the implementation process model by Grol and Wensing [10] coupled with an exemplarian participatory action research approach [11]. The process model of Grol and Wensing is a “how-to-implement” model, based on multiple theories of behavioral change related to individuals, and the influence of social, organizational, and economic context [10]. It consists of five steps:

1. Development of concrete proposal/targets for improvement or change
2. Analysis of current performance, target group, and setting
3. Development/selection of strategies and measures to change practice
4. Development, testing, and execution of implementation plan
5. (Continuous) evaluation and (where necessary) adapting plan

We have used this framework to structure and support planning and execution and evaluation of the implementation strategy and combined it with an exemplarian participatory action research (PAR) approach [11]. According to Baum et al. “PAR seeks to understand and improve the world by changing it” (page 854) [12]. This approach is based on reflection, data collection, and action by the people who will be affected by the intended changes and the researchers with the main goals of understanding and changing the current situation. Research has shown that this type of co-creation, where researchers collaborate with the people who will be affected by the intended changes, is beneficial for the implementation of an innovation in health systems [13, 14]. Because end-users will be involved in the implementation project, they are likely to feel more ownership and responsibility for the innovation. Furthermore, they can reflect on their own actions and they will learn what the success factors are, which in turn will support the sustainable implementation of ALS Home-monitoring and Coaching within their own organization.

**Fig. 2:**
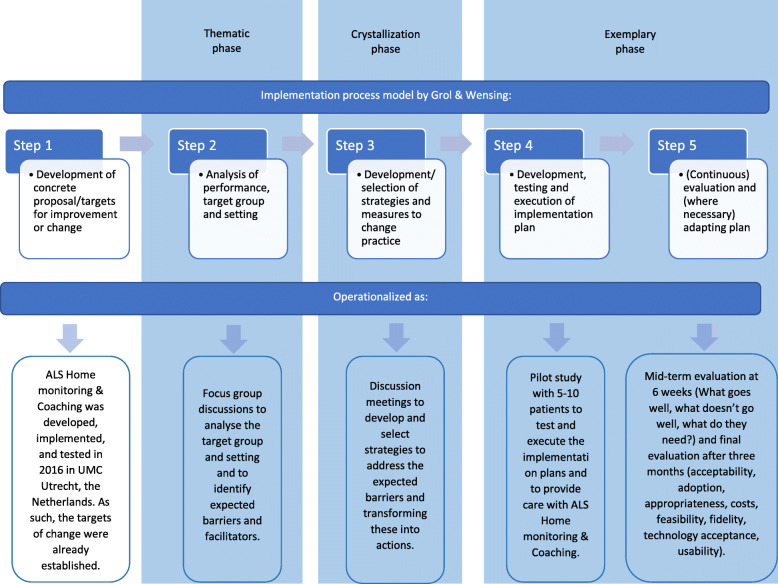
The implementation process

The implementation process is divided in to three main phases: thematic phase, crystallization phase, and exemplary phase. How these phases relate to the five steps of the process model by Grol and Wensing and how each phase and step is operationalized in our project is illustrated in Fig. 2.

The thematic phase is a diagnostic phase. In this phase, we will organize focus groups with each project team to make an inventory of the current situation, the target group, the targets for change, and to analyze the barriers and facilitators the project teams expect to encounter when implementing ALS Home-monitoring and Coaching within their own organization. The Theoretical Domain Framework (TDF) was used to develop the topic guide for the focus groups. The TDF combines 33 behavioral change theories relevant to implementation and divides the constructs from those theories into 14 domains that explain the potential determinants of behavior [15–18]. The topic guide includes a set of semi-structured, open-ended questions and prompts asking the project team about among others their knowledge, skills, beliefs about consequences, and the environmental context regarding ALS Home-monitoring and Coaching and how those domains may influence the implementation within their own organization. The focus groups will be recorded and all audio recordings will be transcribed verbatim and anonymized. The researcher(s) will analyze the data from focus group transcripts thematically, using both a deductive and inductive approach, to order the expected barriers and facilitators [19]. This will be summarized in a report that will be returned to the project team for comments or corrections.

In the crystallization phase, the identified barriers and facilitators will be discussed with the project team. The project team will be invited to develop a concrete action plan with strategies for how to resolve each barrier. There are two strategies that will be included in each action plan, i.e., (1) adapting the monitoring and care protocol to local factors, and (2) training of the healthcare coach. Although the monitoring and care protocol is based on existing (multidisciplinary) guidelines and thus aspires a uniform working method, each team is allowed to make adjustments to improve the applicability within their own ALS care team. Those changes can be related to, e.g., the referral to other disciplines in- and outside the ALS care team and the provision of information to their patients. The healthcare coaches will be trained by the research team with assistance of an experienced healthcare coach of the initial location (the University Medical Centre Utrecht).

The project teams will be allowed at least 4 weeks to realize the preconditions necessary for the exemplary phase. In the exemplary phase, the teams will have approximately 3 months to test and execute their implementation plan. Each team will include 5-10 ALS patients with whom they will try ALS Home-monitoring and Coaching in a pilot study. After 3 months, participating patients and their healthcare providers will be asked to participate in the evaluation study and, if willing, they will be sent a digital survey in order to assess implementation outcomes and user experiences. The research team will evaluate the implementation and user experiences and will organize feedback meetings with the ALS care teams to show and discuss the results, after which the ALS care teams can adapt their implementation plan where necessary before providing this type of care to all their patients with ALS.

### Evaluation

#### Midterm evaluation

For a successful implementation, it is important to evaluate regularly and adapt plans when necessary. Therefore, we will stay in touch with the teams on an informal basis to monitor how the implementation process is going. At approximately 6 weeks after the start of the pilot (exemplary) phase, we will plan a formal midterm evaluation with each team to discuss by phone: (1) What is going well?, (2) What is not going well yet?, and (3) What do they need? This can trigger the teams to slightly tweak their implementation strategies if necessary.

#### Final evaluation

To evaluate the success of the implementation, we will use the evaluation framework by Proctor et al. [20] combined with the telemedicine technology acceptance model by Chau et al. [21] to assess the technological acceptance and the System Usability Scale (SUS) to assess the usability of the app and platform. Data will be collected with an online survey that will be distributed among patients and healthcare providers of the ALS care team approximately 3 months after the patients having started with ALS Home-monitoring and Coaching (See Additional files 1 and 2). In addition, app measurements, registration systems, and field notes will be used to collect relevant data (detailed below).

##### Implementation outcomes

The evaluation framework by Proctor et al. consists of the following implementation outcomes: acceptability, adoption, appropriateness, costs, feasibility, fidelity, penetration and sustainability [20]. The costs, penetration (“the integration of a practice within a service setting and its subsystems” Proctor et al. 2011, page 70), and sustainability (“the extent to which a newly implemented treatment is maintained or institutionalized within a service setting’s ongoing, stable operations” Proctor et al. 2011, page 70) will not be part of the final evaluation of this implementation project because the follow-up period is too short to be able to measure those three implementation outcomes. Nevertheless, we hope we can evaluate the costs, penetration, and sustainability of ALS Home-monitoring and Coaching sometime in the future. The other implementation outcomes are described below.

#### Acceptability

Acceptability is the perception of the patients and healthcare providers that various aspects of ALS Home-monitoring and Coaching (e.g., content, complexity, comfort, delivery and credibility) are to their satisfaction, based on their own experience and from their own point of view. Acceptability will be assessed by questionnaire with questions such as “How satisfied are you with the care you’ve received as part of ALS Home-monitoring and Coaching?” and “How satisfied are you with the ALS app?”. See Additional files 1 and 2 for the full questionnaires.

#### Adoption

Adoption of ALS Home-monitoring and Coaching, or the uptake, can be assessed at two levels, i.e., organizational and patients’ level. First, to assess the adoption at the organizational level, we will assess how many ALS care teams were approached, how many teams intended to participate, and how many teams completed the full implementation process. Second, to assess adoption at the patients’ level, each team will keep track of the total number of patients approached to participate in ALS Home-monitoring and Coaching, how many patients intended to participate and how many patients stopped prematurely.

#### Appropriateness

Appropriateness relates to the extent that patients and healthcare providers feel ALS Home-monitoring and Coaching fits within their setting, how compatible it is with their current work/care methods and whether they find it relevant. It will be assessed by questionnaire with statements such as “The way I receive care with ALS Home-monitoring and Coaching meets my care needs” and “An e-health concept of care such as ALS Home-monitoring and Coaching fits within my organization” with answer categories ranging from totally agree to totally disagree.

#### Feasibility

The feasibility of ALS Home-monitoring and Coaching, according to patients and healthcare providers, tells something about the extent to which they think the program can successfully be used within their specific context [20], in other words, whether it is suitable for everyday use. We will assess the feasibility by questionnaire with statements such as “The use of ALS Home-monitoring and Coaching quickly becomes routine” and “I often forget to open and use the ALS app” with answer categories ranging from totally agree to totally disagree.

#### Fidelity

The degree to which ALS Home-monitoring and Coaching has been implemented in each participating rehabilitation center as it was intended is defined as fidelity [20, 22]. We will keep track of any deviations from the original implementation plans that may happen, as well as any possible changes to the concept of ALS Home-monitoring and Coaching. Adherence is part of fidelity, and in our study, this is defined as the extent to which the healthcare providers used the platform, how often they completed the planned monitoring sessions, how many patients completed the pilot phase, and to what extent patients completed the number of measurements that were agreed upon at the start. This data will be downloaded from the platform.

#### Technology acceptance

Technology acceptance can be interpreted as the psychological state of a person regarding their voluntary or intended use of a certain technology [23]. To further examine the technology acceptance of ALS Home-monitoring and Coaching by patients and their healthcare providers, we will use the (revised) telemedicine technology acceptance model by Chau et al. [21]. This model is based on the Technology Acceptance Model (TAM) and the Theory of Planned Behavior (TPB) [24, 25]. According to the TAM, someone’s intention to use a particular technology depends on attitude, perceived usefulness, and perceived ease of use [21, 25]. According to the TPB, there is another important determinant that influences behavioral intention, i.e., perceived behavioral control [21, 24]. The technology acceptance model of Chau et al. used both models as a theoretical basis and theorizes that an individual’s technology acceptance is influenced by factors related to the (1) individual context (i.e., attitude, perceived technology control), (2) the technological context (i.e., perceived usefulness, perceived ease of use), and (3) the implementation context (i.e., compatibility with current work practices, peer influence). Chau et al. developed several question items to assess the aforementioned constructs, which we slightly adapted to the specific context and translated to Dutch. Additional question items to further operationalize the constructs were self-developed and adapted from literature [23]. To assess patients’ and healthcare providers’ technology acceptance of ALS Home-monitoring and Coaching, we have added all those question items to the online evaluation surveys. See Additional files 1 and 2 for the full questionnaires.

#### Usability (SUS)

To examine the usability of the ALS Home-monitoring and Coaching platform, we will use the Dutch version of the System Usability Scale (SUS) [26, 27] and several self-developed questions. See Additional file 2 for the full questionnaire. The SUS is a simple Likert scale that is often used to provide a global view of subjective assessment of usability. It consists of 10 items and answer categories range from [1] totally disagree to [5] totally agree. A total score will be calculated and can range from 0 to 100, with higher scores indicating a favorable usability.

### Analyses

We will use a mixed methods approach to evaluate the implementation process and outcomes.

Qualitative methods will be used to understand the specific contexts, the expected barriers and facilitators, which adaptations were made to ALS Home-monitoring and Coaching to fit each context, which changes were made to the original implementation plan (fidelity), and which implementation strategies were used. Data from focus group transcripts, open-ended survey questions, and field notes will be thematically analyzed, taking both a deductive and inductive approach [19]. We will start with a deductive approach using the Theoretical Domain Framework to inform the initial coding scheme and we will further explore unanticipated themes using an inductive approach [15–18].

Quantitative methods (descriptive statistics, e.g., frequencies, means, medians) will be used to assess the following outcomes: acceptability, adoption, appropriateness, feasibility, adherence, technological acceptance, and usability.

### Timeline

**Fig. 3:**
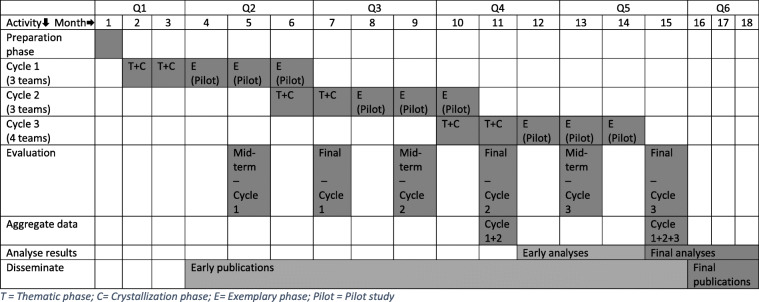
Project timeline

The duration of the total project is 18 months. The individual implementation process for each participating team takes 5–6 months. The implementation takes places in three cycles, with 3–4 teams per cycle. The start of the implementation process of one cycle is 3–4 months after the start of the previous cycle. The implementation process is divided in three main phases: thematic phase, crystallization phase, and exemplary phase (as described above). The thematic and crystallization phase together span approximately 2 months and the exemplary phase will be 3 months. The midterm evaluation within each cycle will take place approximately 6 weeks after the start of the exemplary phase. The final evaluation of each cycle will take place at the end of the exemplary phase. See Fig. 3 for a schematic representation of the project timeline.

## Discussion

E-health innovations are becoming increasingly important in healthcare, but it is imperative to learn how optimal uptake of such innovations in daily clinical practice can be ensured in order to provide the best care possible to patients tailored to their needs. To be able to learn this and to explain the reasons how and why an implementation succeeds or fails, a thorough theoretical approach to implementation is imperative [28]. By using implementation theories to structure and support planning, execution and evaluation of the implementation of ALS Home-monitoring and Coaching, the current study adds to the literature on identifying factors contributing to implementation success and identifying strategies that will lead to a more successful implementation. More specifically, this research will enhance our understanding of how to successfully implement e-health innovations in multidisciplinary care in rehabilitation settings.

In addition, this implementation study will lead to quality improvement of the care for patients with ALS in the ten participating centers, especially because the care will be more tailored to the patients’ needs. The results of this study will also be useful for the remaining ALS care teams in the Netherlands, and beyond, because a comprehensive implementation guide based on the aggregated results and experiences of the 10 participating centers will be written and made available on the ALS website (https://www.als-centrum.nl/). The implementation guide can be used by every ALS care team to implement or scale up ALS Home-monitoring and Coaching within their own setting. Moreover, the innovation and the acquired knowledge regarding the implementation may be applicable for other diagnoses as well.

The number of e-health innovations has been increasing rapidly since the term e-health was first introduced in 1999 [29, 30], but this is the first time an e-health innovation is being implemented in a nationwide ALS care network [31]. E-health innovations that can monitor disease progression of ALS patients have great potential to better tailor the care to the needs of individual patients and to lessen the burden for patients of having to travel to the clinic and attend long and exhausting clinic days, but, nevertheless, we face several challenges (personal, organizational and societal) with implementing ALS Home-monitoring and Coaching. One of the challenges is the multidisciplinary nature of the ALS care teams with many people involved in the care of the ALS patients, including physiatrists, physical, speech, and occupational therapists. This means that a large number of people will be affected by the innovation. Another challenge for this implementation is that the ALS care teams are situated in different rehabilitation settings (i.e., academic hospitals, regional hospitals, rehabilitation centers) with each their own and distinct organizational and financial structures and resources. A third challenge is the fact that the implementation takes place amidst the COVID-19 pandemic that the world is currently facing. As a result of the pandemic, there are now new barriers for patients with ALS to receive in-clinic multidisciplinary care, in addition to the difficulties patients already face to travel to and attend in-clinic visits for example due to mobility limitations or because of the distance to the ALS center [3, 32]. The COVID-19 pandemic has increased the sense of urgency to develop and implement e-health innovations, so patients with ALS can be monitored remotely and are able to receive the care they need even when they are not able to visit the multidisciplinary care team in person [32]. However, the COVID-19 pandemic has presented us with some practical challenges for the implementation of ALS Home-monitoring and Coaching, one of these being unable to conduct the focus groups with all participants being present in the same room.

### Strengths and limitations

The use of implementation theories to structure and support planning, execution, and evaluation of the implementation of ALS Home-monitoring and Coaching is one of the main strengths of this study. Another strength is the participatory action research approach. Patients and their caregivers have not only been involved in the development of ALS Home-monitoring and Coaching [5, 9], but they are also involved in each step of the implementation process. They will be the end-users of the innovation and they can provide a unique and complementary perspective based on their own expertise and experiences [33]. Also other people who will be affected by the intended changes, such as physiatrists, allied health professionals, managers, and schedulers will be involved in the implementation process. Research has shown that this type of co-creation is beneficial for the implementation of an innovation in health systems [13, 14]. Because end-users will be involved in the implementation project with this bottom-up approach, they are likely to feel more ownership and responsibility for the innovation which will empower them to achieve change [34]. Furthermore, the participatory action research approach will facilitate the teams with the tools to reflect on and learn from their own actions and the success factors; information and experience that they can use for scaling up ALS Home-monitoring and Coaching within their own setting. We will use both qualitative (e.g., focus groups) and quantitative (e.g., online surveys, app data) research methods to evaluate the implementation, which is another strength of this study.

There are also a few limitations that need to be taken into account. ALS Home-monitoring and Coaching will be implemented following the same implementation strategy (as described in this paper) in each of the ten participating centers and as such no comparisons can be made between different implementation strategies. Also, we have chosen to implement the platform as a stand-alone platform, despite knowing that integration with the existing electronic patient records would probably facilitate sustainable implementation [35]. In the Netherlands, multiple electronic patient record systems are being used in rehabilitation. Consequently, there is no one-size-fits-all approach to integrate the ALS Home-monitoring and Coaching platform with the electronic patient record systems of the ten participating ALS care teams. The technical integration was deemed too challenging to be part of this implementation project. It will need to be addressed in the future. The high costs that are involved in building and maintaining e-health platforms are often important barriers for sustainable implementation in daily clinical practice [31]. Although the costs for the ALS Home-monitoring and Coaching platform for the ten participating centers are covered until December 2021 by the research grant, the financial coverage is not yet guaranteed for the future. This uncertainty might prevent the participating centers to fully commit to the changes working with ALS Home-monitoring and Coaching will require of them. Meanwhile, we will explore options for structured and more permanent funding by health insurance providers, patient organizations, and/or hospitals or rehabilitation centers.

## Conclusion

It is anticipated that by using implementation theories coupled with a participatory action research approach this study will provide inside in factors influencing implementation outcomes and strategies that can be used to overcome barriers. More specifically, this study will enhance our understanding of how to successfully implement e-health innovations in multidisciplinary care in rehabilitation settings. This knowledge may support other implementation initiatives within healthcare settings.

## Data Availability

Not applicable.

## Abbreviations

ALS: Amyotrophic lateral sclerosis
ALSFRS-R: Amyotrophic Lateral Sclerosis Functional Rating Scale-Revised
*N*: Sample size
PAR: Participatory action research
PLS: Primary lateral sclerosis
PMA: Progressive spinal muscular atrophy
SUS: System Usability Scale
TAM: Technology Acceptance Model
TDF: Theoretical Domain Framework
TPB: Theory of Planned Behavior
